# Bichloride of Mercury in Dysentery and Diarrhea

**Published:** 1880-08

**Authors:** 


### BICHLORIDE OF MERCURY IN DYSENTERY AND DIARRHEA.

THE use of *bichloride of mercury in dysentery and diarrhea*, in somewhat minute doses, has been tested by Dr. Reed. "It has been found particularly valuable in those forms of chronic diarrhea characterized by dysenteric symptoms, such as the presence of mucus or blood in the stools, with or without tenesmus." The notes of four cases thus treated are given.

